# Collective escape waves provide a generic defence against different avian predators

**DOI:** 10.1098/rsos.241055

**Published:** 2025-03-12

**Authors:** David Bierbach, Juliane Lukas, Luis Gómez-Nava, Fritz A. Francisco, Lenin Arias-Rodriguez, Stefan Krause, Korbinian Pacher, Yunus Sevinchan, Pawel Romanczuk, Jens Krause

**Affiliations:** ^1^ Department of Biology and Ecology of Fishes, Leibniz-Institute of Freshwater Ecology and Inland Fisheries, Müggelseedamm 310, Berlin 12587, Germany; ^2^ Faculty of Life Sciences, Albrecht Daniel Thaer-Institute of Agricultural and Horticultural Sciences, Humboldt-Universität zu Berlin, Invalidenstrasse 42, Berlin 10115, Germany; ^3^ Cluster of Excellence ‘Science of Intelligence’, Technical University of Berlin, Marchstrasse 23, Berlin 10587, Germany; ^4^ Department of Biology, Institute for Theoretical Biology, Humboldt-Universität zu Berlin, Philippstraße 13, Berlin 10115, Germany; ^5^ Department of Biology, University of Massachusetts Boston, Boston, MA, USA; ^6^ División Académica de Ciencias Biológicas, Universidad Juárez Autónoma de Tabasco, Villahermosa 86150, Mexico; ^7^ Department of Electrical Engineering and Computer Science, Lübeck University of Applied Sciences, Lübeck 23562, Germany; ^8^ Bernstein Center for Computational Neuroscience Berlin, Philippstraße 13, Berlin 10115, Germany

**Keywords:** predation, collective behaviour, fish, birds, extremophile fishes, escape behaviour

## Abstract

In many animal species, collective behaviours can be explained by a simple set of interaction rules. It is an intriguing question whether this generality at the level of mechanism also translates into generality at the level of function. Assuming that collective behaviour provides antipredator benefits for the partaking individuals, we ask whether the same collective behaviour provides protection against different predators in general. We investigated this question in sulphur-adapted fishes in their natural habitats in Mexico. Here, fish schools are frequently attacked by many different bird species and fish respond with synchronized and often repeated collective diving behaviour (escape waves). We found all bird species to wait longer until they attacked as they encountered more waves, both before they launched their first attack (pre-attack) and between subsequent attacks (post-attack). Post-attack, all bird species triggered similarly high numbers of waves while species differed in the number and the interval between the waves they triggered pre-attack. Through simulated bird arrivals, we confirmed that birds in the pre-attack context could be perceived as less threatening or completely overlooked, depending on their size, colouration and contrast to the background. We argue that the generality in the fish’s collective response as well as the similarity in effect on the different birds’ hunting behaviour might be explained by waves targeting a weakness in the visual processing ability common to different predators.

## 1. Introduction

Many studies have addressed the antipredator benefits of group living [1]. In studies on fish schools, for example, this is typically tested by investigating predator capture success in relation to school size with larger schools providing more antipredator benefits than smaller ones [2–5]. However, the schooling behaviour itself was never quantified in these experiments and to demonstrate its adaptive value, one would need to increase the number of individuals with and without social coupling (i.e. schooling behaviour or other types of collective behaviour) between them to exclude the possibility of a pure numbers effect that reduces predator success through confusion without schooling behaviour [6]. This problem was partially addressed by Ioannou *et al*. [7] who showed in a laboratory study that live predators selected for proto-collective behaviours in virtual prey, as the live predatory fish in their experiments attacked predominantly lone virtual prey items displayed on a screen and to a lesser extent those moving together. Interestingly, prey that moved coordinated and aligned with their neighbours (e.g. that were schooling) received less attacks as compared with unpolarized shoals. This result suggests that it was indeed the social coupling and not the sheer number of individuals that provided antipredator protection. However, this hypothesis needs further support from real predator–prey interactions in the wild.

There is a growing body of studies which shows that collective behaviours, such as waves and spirals that are emergent patterns of the interactions between individuals, may have adaptive properties in themselves. In giant honeybees, *Apis japonica*, and starling flocks, *Sturnus vulgaris*, it was shown that the occurrence of spirals and waves was connected to predator attacks [8,9]. Doran *et al*. [10] went a step further and demonstrated a causal link between the occurrence of waves produced by sulphur mollies (*Poecilia sulphuraria*) and a reduction in capture probability in great kiskadees, *Pitangus sulphuratus*, attacking the fish schools. Besides these few studies that tested for the adaptive significance of actual collective behaviour, prey’s collective responses were usually investigated in relation to only a single predator species. As prey is usually in the focus of many different predator species, this leaves open the question of whether these collective behaviours represent a generic mechanism that protects against various different predator species.

**Figure 1.**
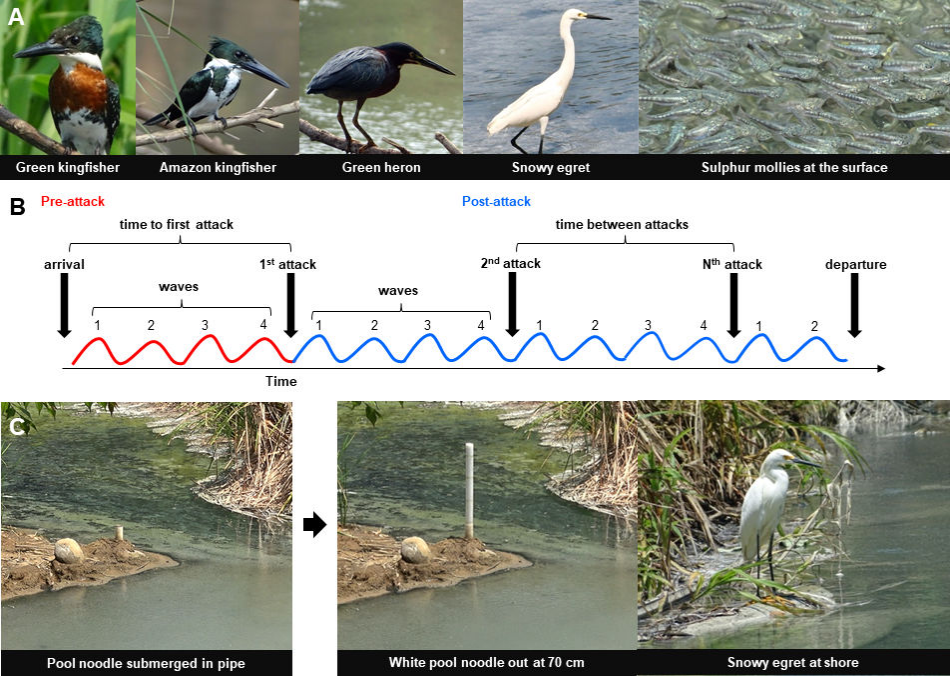
The sulphur fish system in Southern Mexico. (A) The main avian predators of the sulphur fishes. (B) Definition of pre- and post-attack contexts. Waves and times to attack were counted either from the arrival of the bird on-site to the first attack (pre-attack context) or from attack to attack (post-attack context). (C) Experimental simulation of arriving birds with pool noodles that emerge from the ground to specific heights. Shown is a white noodle lifted to 70 cm. This way the arrival of a snowy egret is mimicked. More information on the stimuli creation is presented in electronic supplementary material, figure S1 and video S4.

We investigated this question in a predator–prey system in Mexico. Here, two sulphur-adapted fish species (the sulphur molly, *P. sulphuraria* as well as the widemouth gambusia, *Gambusia eurystoma*) are the only fish species inhabiting sulphidic spring complexes. Due to the high H_2_S levels, these rivers are almost free of dissolved oxygen [11–13] and fishes spend most of their time at the water surface in schools sometimes comprising thousands of individuals per square metre [10]. These aggregations attract many bird species that represent the only fish predators in this system (figure 1A, [13–15]). In response to an approaching bird (termed hereafter ‘pre-attack context’) or after a bird’s attack (‘post-attack context’, see figure 1B), fish dive down in a staggered fashion. As the fish start their diving close to the water surface, they break the surface shortly with their caudal fins which is resulting in large collective surface waves travelling across the schools. The fish then quickly resurface 3−4 s later and often dive again repeatedly (see electronic supplementary material, video S1 [10,15]).

In a first step, we filmed bird attacks on fish schools in different locations along the sulphidic river habitat over several years (2016−2019 and in 2023). We established (i) whether the waving was effective in terms of delaying attacks and/or reducing capture success focusing on four bird species (green kingfisher, Amazon kingfisher, green heron and snowy egret) which differed in size, colouration and attack strategy [15]. Furthermore, we recorded (ii) whether the waving response of the fish schools in terms of number and time between waves varied with the predator species.

In a second step, we experimentally investigated selected aspects of the predator–prey interaction. We simulated the arrival of a predator on the river bank (pre-attack context) to test the hypothesis that the waving response of fishes increases with the size and also colouration of the predator stimulus. We identified the pre-attack context as particularly interesting because this is where most of the variation in waving behaviour in response to different natural predators had occurred (see §3). We presented the fish schools with stimuli that covered the observed size range in bird predators as well as the extremes of the colour/contrasts (e.g. snowy egrets are white and green heron dark brown) by using grey and white pool noodles that emerged from the ground to different lengths (figure 1C, electronic supplementary material, figure S1 and video S4).

## 2. Methods

### 2.1. Study system

We studied the collective behaviour of sulphur-adapted fishes in sulphidic springs in Southern Mexico (17°33′ 10.1″ N, 92°59′ 49.7″ W, Hacienda del Azufres near City of Teapa, Tabasco). Only two fish species, the sulphur molly and the widemouth gambusia, have colonized the hydrogen sulphide-rich springs of our study site [16,17] with mollies being more common and more active regarding the waving behaviour [18]. We thus refer to sulphur molly schools within this manuscript although it is possible that widemouth gambusia were present at lower abundance. Besides the toxicity of H_2_S in the water, these aquatic habitats are also almost free of dissolved oxygen and as a behavioural adaptation to the hypoxia, fish spend large proportions of their time shoaling directly below the water surface [13,19,20]. These fish aggregations attract large numbers of fish-eating bird species and thus experience frequent encounters with predators [10,13–15]. Birds were the only predators feeding on the sulphur mollies (electronic supplementary material, videos S2 and S3) and no piscivorous fish or mammals were observed. In response to approaching and/or attacking birds, the fish briefly dive down and when their tails touch the water surface ripples can be seen from the outside as a distinct wave at the surface (electronic supplementary material, video S1).

### 2.2. Natural observations of bird attacks and fish waves

Bird attacks on fish schools were recorded in many different locations along the river with video cameras (Sony HD Handycams). For our study, we focused on four bird species: green kingfisher, *Chloroceryle americana* (body length 21.7 cm, weight 47 g, wingspan 9.3 cm)*,* Amazon kingfisher, *Chloroceryle amazona* (30 cm, 140 g, 50 cm), green heron, *Butorides virescens* (41−46 cm, 240 g, 64−68 cm) and snowy egret, *Egretta thula* (56−66 cm, 370 g, 100 cm), that were common and represented the greatest possible variation in size, appearance and hunting strategy (for details see figure 1A and [15,21,22]). Herons and egrets were often found wading along shore or in shallow water, where they searched for prey. Early detection of these predators is likely dependent on their size and movement. Snowy egrets were often seen running, during which they flap their wings, causing lots of fish movement and water waves. In stark contrast, green herons remain almost motionless usually on the river bank before they ambush their prey. Prior to an attack, kingfishers often perched near or above the water, where they were inconspicuous against the background vegetation. When they launched an attack, they flew in a direct line for their prey. As they closed the distance to the water, kingfishers folded back their wings, thus exposing their white underbelly. Like a projectile, kingfishers plunge-dive into the water after their prey, fully submerging their body and thus greatly disturbing the water surface upon impact.

We recorded birds from their arrival on a perch or on the river bank until they flew off and used a time stamp approach through which we noted to the second the time of each attack as well as the time of each wave. As distinct fish waves emanating from bird arrivals or attacks are well visible from the outside (see electronic supplementary material, videos S1 and S3), this enabled us to calculate the following variables describing the birds’ and the fishes' behaviours during each attack bout (=successive number of attacks launched by the same bird towards the same fish school until leaving the site): (i) ‘time to first attack’: from the arrival of a bird until it launched the first attack; (ii) ‘attack intervals’: time between successive attacks; (iii) ‘number of waves’: count of successive waves before the initial attack (pre-attack) or after subsequent attacks (post-attack) until the next attack was launched or the bird flew off; and (iv) ‘wave interval’: time between two successive waves (separately for both pre- and post-attack occurring waves). Note that for each attack bout that could comprise up to 13 attacks, pre-attack waves were only considered before the first attack, thus leading to a natural mismatch between sample sizes of attacks analysed in pre- and post-attack contexts.

We were unable to identify birds at the individual level across different attack bouts. However, from spot-checks during river transects and territorial disputes between birds, we know that multiple pairs of each species were breeding and hunting in the vicinity (in some cases with their offspring) with a possibility of others visiting the area. Furthermore, we carried out our study along large sections of the river and over several years, which is likely to include some generational turnover, and are therefore confident that a good sample of the predator population was studied.

#### 2.2.1. Statistical analysis

In our first analysis (model 1a, see electronic supplementary material, data S1 for any statistical model outputs), we used a linear mixed effects model (Poisson error distribution with log link function) to compare the number of waves triggered (dependent variable) among all bird species (four species, independent variable) and attack context (pre- and post-attack, independent variable). We further included the interaction term between these factors and used attack bout ID as a random factor. For time between waves (interval between successive waves; model 1b), we used a similar model (please note that the Amazon kingfisher was included in the model although never more than one wave was triggered in the pre-attack context and thus no wave intervals were included for this species in the pre-attack context).

The second analysis focused on the consequences of waves on predator hunting success. Here, we compared overall capture success (binary variable: 1 = fish caught, 0 = no fish caught in attack) of each species in the pre- and post-attack context using χ² tests. Furthermore, we compared in GLMs separately the time until the first attack (model 2a; sqrt-transformed) as well as the time between subsequent attacks among bird species (model 2b; sqrt-transformed).

In a third set of analyses, we used linearized models to see whether the number of waves seen before the attack had an effect on both capture success and time to launch the attack. In model 3a, we used time to first attack (sqrt-transformed) as the dependent variable and included species ID and number of waves before attack (and their interaction term, wave number as sqrt-transformed) as fixed factors. Similarly, for the pre-attack context, we used capture success of the first attack as the dependent variable and included species and number of waves before attack (and their interaction term) as fixed factors in model 3b. Analogously, we analysed the effect of wave number seen on capture success and time to attack in the post-attack context with mixed models and included attack bout ID as a random factor (models 4a,b). Note that for models 3 and 4, only attacks that triggered more than one wave (repeat waves) were considered. For the analysis of time to attack, we only considered cases where birds waited less than 300 s (5 min) to attack to make sure we did not inflate the analysis with cases where birds were not actively hunting (*n* = 2 cases removed).

### 2.3. Experimental stimulus presentation for pre-attack context

We experimentally investigated selected aspects of the predator–prey interaction by simulating the arrival of a predator on the river bank (see electronic supplementary material, data S1 for sample sizes). We presented fish schools with stimuli that covered the observed size range in bird predators as well as the extremes of the colour/contrasts (e.g. snowy egrets are white and green heron dark brown) by using dark grey and white pool noodles of 10, 30, 50, 70 and 90 cm (see figure 1C for a depiction of the setup as well as electronic supplementary material, figure S1 and video S4). We repeated our experiments at five different river locations and depending on shoreline characteristics like steepness and rockiness, the pool noodles either emerged vertically from the ground or extended horizontally from the river bank over the water. This was done by having the pool noodle inside a PVC pipe buried 1 m deep in the group or laid flat into the shore vegetation. The pool noodle was initially covered completely inside the PVC pipe that had a slightly larger diameter. We pulled the noodle out by attaching a thin fishing line to the bottom end of the noodle and rolling it up (see electronic supplementary material, figure S1 and video S4). For each location, we simulated two different background scenarios commonly experienced by the fish: in one treatment, the noodles appear in front of the brown river bank or in a second treatment with the sky as background (in one location, only measurements against the sky were possible). The presentation of the different noodle sizes, colours and backgrounds was organized in batches each comprising one colour and background and all five noodle sizes in random order. Within each batch, noodle sizes were presented for 30 s with a 6 min break in-between to avoid fish getting habituated to the stimulations (no effect of the order on the number of waves within batches was detectable *a posteriori*: Pearson’s *r* = −0.017, *p* = 0.7, *n* = 450). We tested the hypothesis that the dark noodles should be more cryptic and trigger less waving against the river bank background but more conspicuous with more waving against the sky compared with the white noodle. From our video recordings, we counted the number of waves initiated by the fish schools at the respective location and measured the time between waves. All experiments were recorded with a Sony 4K Handycam at 25 fps and 4K resolution.

#### 2.3.1. Statistical analysis

We first analysed the number of waves in a mixed model (model 5a, log-transformed, Gaussian error distribution and identity link function) with location ID and batch ID as random factors. As fixed factors, we included stimulus size, stimulus colour and background as well as their interactions. Non-significant interactions were step-wise removed and the final model only contained the interactions between ‘stimulus colour × background’. Analogously, we analysed wave frequencies (model 5b, gamma distribution with log link function). Here, the final model only contained the main effects (see electronic supplementary material, data S1). Statistical analysis was performed in SPSS22 (IBM Inc.) and figures were prepared using Prism (GraphPad Inc.).

## 3. Results

### 3.1. Observational studies on bird–fish interactions

#### 3.1.1. Fish responses towards attacking birds

**Figure 2.**
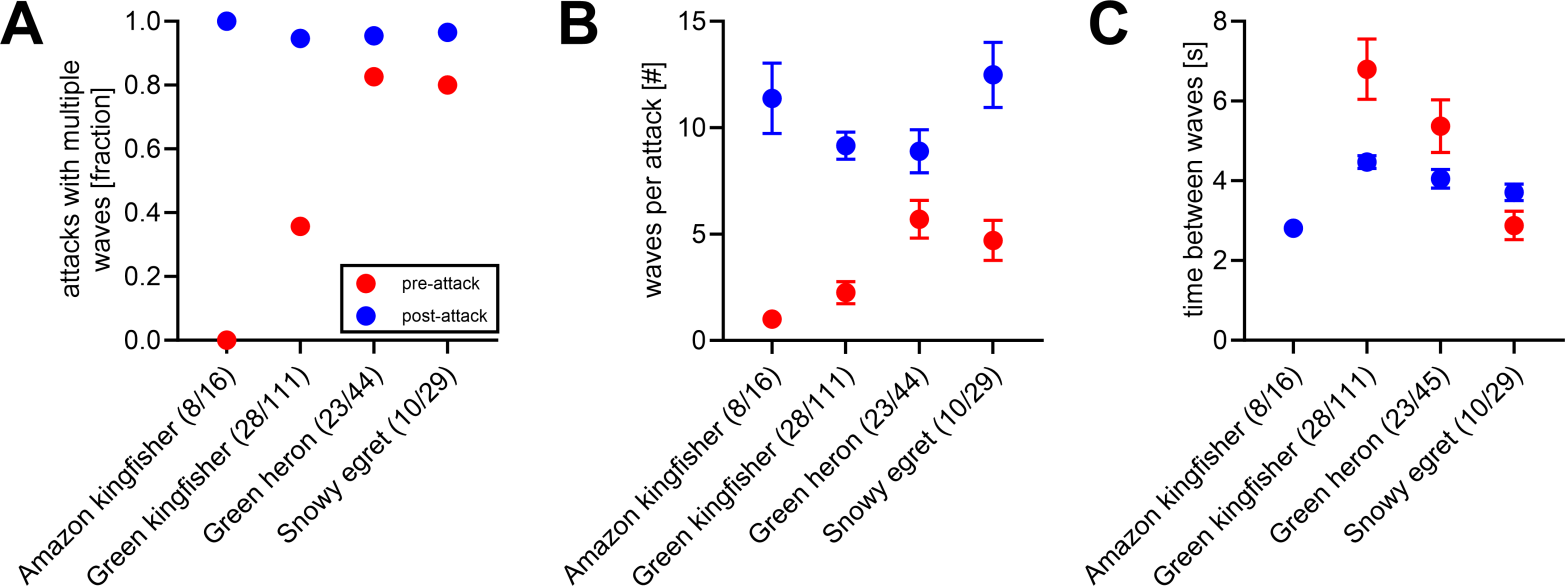
Sulphur-adapted fishes’ collective wave response towards attacks of different bird species. (A) Proportion of bird attacks that triggered more than a single wave before the attack (pre-attack; red) and after the attack (post-attack; blue). (B) Number of waves per attack. This shows the average number of waves that were triggered by an attack before the attack happened (pre-attack; red) and after the attack (post-attack; blue). (C) Time between waves per attack. For (B and C), we show averages along with s.e.m. Sample sizes for pre- and post-attack contexts are given in bracelets behind the bird species name and can be found in electronic supplementary material, data S1.

By analysing a total of 69 attack bouts with 200 attacks and 2200 fish waves triggered, we found that fish performed multiple waves after attacks (post-attack context) for all birds with very high probability (all >95%), but only for herons and egrets, multiple waves were triggered with more than 80% probability on arrival, while kingfishers either never (0% Amazon Kingfisher) or rarely (36% Green Kingfisher) triggered multiple waves before starting their attacks (figure 2A). This difference between pre- and post-attack contexts as well as among different bird species in the pre-attack context can also be seen in the absolute numbers of waves triggered; i.e. on average, more waves were recorded after birds had launched their attacks than in response to their first appearance (model 1a: main effect ‘attack context’: *F*_1,261_ = 46.5, *p* < 0.001, figure 2B). Kingfishers triggered fewer waves before their first attacks compared with egrets and herons (figure 2B). Importantly, similar wave numbers were observed in the post-attack context in all bird species, i.e. after the attacks were launched (figure 2B; interaction term ‘bird species × attack context’: *F*_3,261_ = 6.4, *p* = <0.001). This means that although birds differed in the number of waves they triggered when arriving, they caused fish to perform similar numbers of waves after their attacks.

Among the birds that typically triggered multiple waves (except Amazon kingfisher in pre-attack context), the time between the fish waves in the pre-attack context differed among species but waves showed similar intervals between 3 and 5 s in the post-attack context (model 1b; ‘attack context × species’: *F*_2,221_ = 5.2, *p* = 0.006; ‘bird species’: *F*_3,221_ = 8.8, *p* < 0.001; ‘attack context’: *F*_3,221_ = 4.6, *p* = 0.032; figure 2C). Green kingfishers triggered waves with the longest intervals between them.

#### 3.1.2. Bird behaviour in general and in relation to fish escape waves

**Figure 3.**
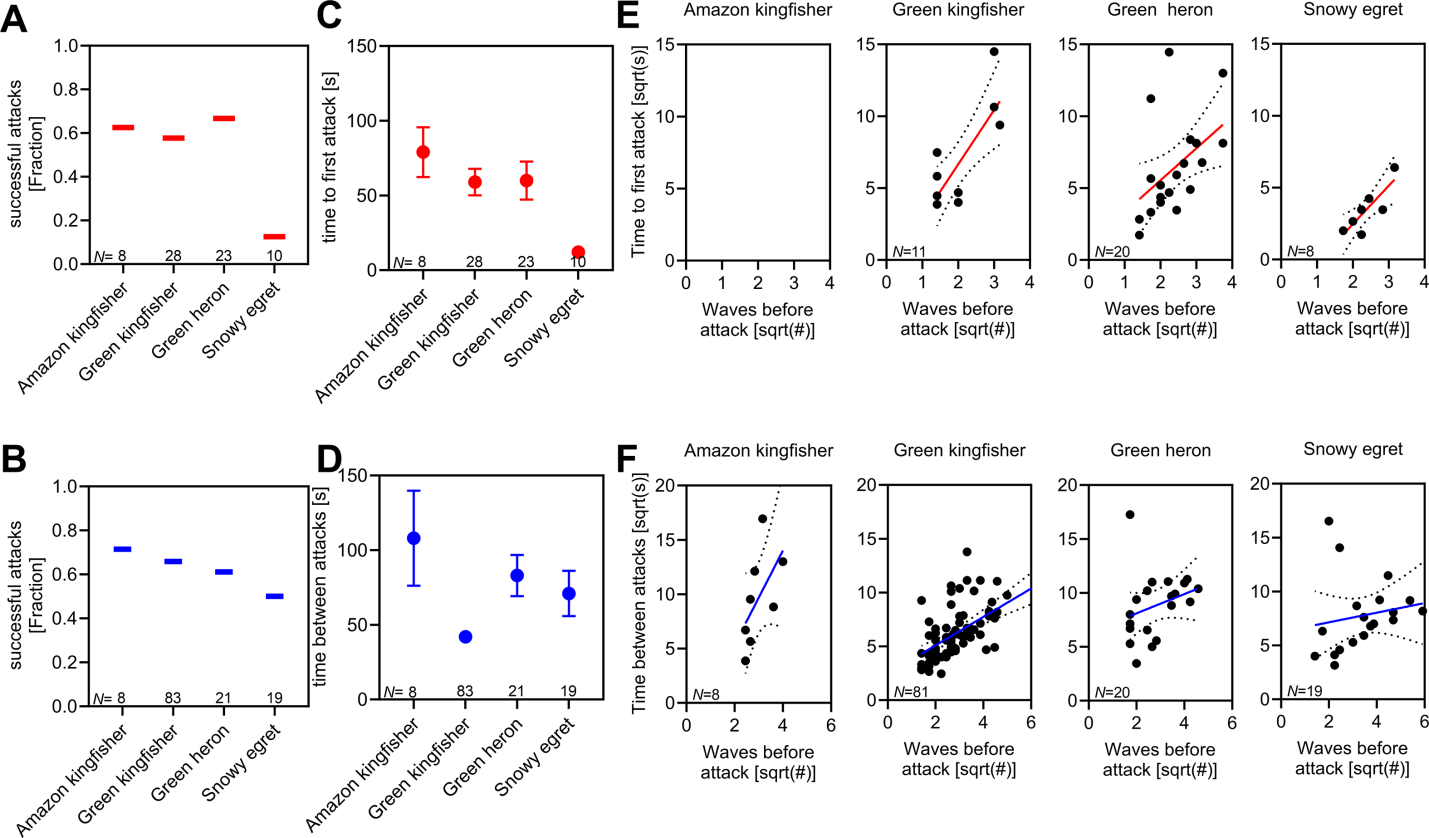
General bird hunting behaviours and effects of fish waves during pre- (red) and post- (blue) attack contexts. (A) Probability to catch a fish in pre-attack context for all four bird species as fraction of successful first attacks. (B) Probability to catch a fish in post-attack context for all four bird species as fraction of successful attacks. (C) Time from arrival to first attack (pre-attack context) and (D) time between subsequent attacks (post-attack context). For panels (A–D), we show averages along with s.e.m. (E) Pre-attack context: time (sqrt-transformed) to a first of subsequent attacks as a function of fish wave number (sqrt-transformed). Please note that we excluded Amazon kingfishers in this figure as these birds only trigger a single wave before their first attack. (F) Post-attack context: time (sqrt-transformed) between subsequent attacks as a function of fish wave number (sqrt-transformed). For panels (E and F), each dot represents a bird attack. Sample sizes are given in the graphs and can be found in electronic supplementary material, data S1.

In general, bird species did not differ in their success when attacking the fish schools (figure 3A,B; χ² tests: pre-attack: χ² = 7.26, d.f. = 3, *p =* 0.064; post-attack: χ² = 1.21, d.f. = 3, *p =* 0.75) but we found birds to differ in the number of launched attacks per bout (KW-ANOVA, *H* = 14.48, *p* = 0.002; *n* = 73) and given the similar capture successes per attack, birds differed in the number of caught fish per bout (*H* = 14.37, *p* = 0.002; *n* = 73) with green kingfishers attacked most often in a bout and thus caught most fish per bout (see electronic supplementary material, figure S2). Bird species differed furthermore in the time until they launched a first attack after arrival on the river bank (model 2a; ‘bird species’: *F*_3,63_ = 10.8, *p* < 0.001; figure 3C) with egrets attacking sooner than the other species. Also, there was a significant difference among species in the time between subsequent attacks (model 2b; ‘bird species’: *F*_3,127_ = 3.4, *p* < 0.02; figure 3D), mostly due to green kingfishers which had the shortest waiting times between attacks.

When we analysed the birds’ response in relation to the fish waves, we found that regardless of the bird species, the more waves the fish produced, the longer it took the birds to either launch their first attack (pre-attack context; model 3a: ‘waves before attack’: *F*_1,32_ = 27.1, *p* < 0.001; analysis excluding Amazon kingfishers, figure 3E) or until they attacked the next time (post-attack context; model 4a: ‘waves before attack’: *F*_1,120_ = 11.9, *p* < 0.001; no significant interaction terms between bird species and number of waves in models 3a and 4a; figure 3F). Capture probability was not affected by wave number in either attack context (models 3b and 4b, see electronic supplementary material, data S1). Also, time between waves was neither linked to the time to launch attacks nor to the birds’ probability of catching a fish when attacking (results not shown).

### 3.2. Experimental work on stimulus size, colour and contrast

**Figure 4.**
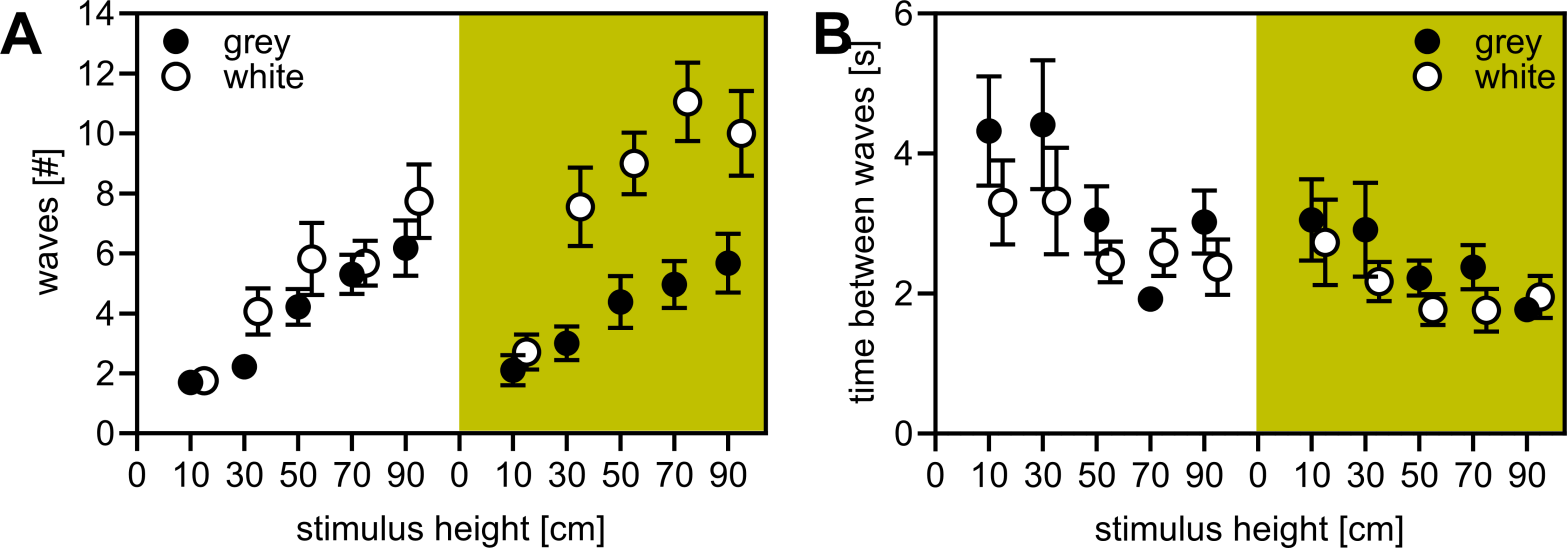
Experimentally induced pre-attack waving response of sulphur-adapted fishes. Shown are (A) number of waves and (B) time between waves towards differently sized grey and white stimuli in front of the open sky (white background) or the river bank with dense vegetation (green background). We depict means with s.e.m. In total, 469 stimuli were analysed, and sample sizes per treatment level can be found in electronic supplementary material, data S1.

Given that pre-attack waving of fish was less pronounced in response to small kingfishers as compared with larger egrets and herons (see figure 2), we experimentally investigated the hypothesis that the waving response of fish increases with the size and colouration of a predator by simulating predator arrival on the river bank using white and grey pool noodles of different sizes. The number of fish waves strongly increased with stimulus size (model 5a; ‘stimulus size’: *F*_4,452_ = 80.7, *p* < 0.001, figure 4A). Furthermore, the waving response showed an interaction effect between ‘stimulus colour × background’ (*F*_1,452_ = 7.4, *p* = 0.007) suggesting that it was dependent on the strength of contrast. Wave responses towards the grey stimuli were background-independent while the white stimuli triggered more waves when in front of dark versus light backgrounds (figure 4A). Overall, more waves were produced in response to white stimuli compared with grey ones (significant main effect of ‘stimulus colour’ *F*_4,452_ = 17.4, *p* < 0.001; and ‘background’, *F*_4,452_ = 5.4, *p =* 0.02). Wave intervals decreased with larger stimuli (model 5b; *F*_4,263_ = 6.7, *p* < 0.001, figure 4B) and were shorter for white stimuli (*F*_1,263_ = 5.7, *p =* 0.018) and when presented in front of the darker background (*F*_1,263_ = 16.7, *p* < 0.001).

## 4. Discussion

### 4.1. Collective waving behaviour is effective against many predators

Fish shoals responded with repeated waves when being attacked by each of the four piscivorous bird species under investigation. Upon arrival, birds that stayed in close proximity to the water (egrets and herons) caused repeated waves as well while both species of kingfishers either never or only rarely caused repeated waves in this pre-attack context. These repeated waves provided antipredator protection in terms of longer waiting times in both pre- and post-attack contexts against all predator species. The relationship between the time birds waited before their first attack or between subsequent attacks and wave number and wave intervals is correlational in this study. However, we build on previous research [10] which showed that the fish waves are causally responsible for a prolongation of the waiting times in birds in our study system.

The fact that a repeated waving response by fish reduced attack frequency in bird predators from three different orders (*Passeriformes* [10], *Coraciiformes* and *Pelecaniformes*) suggests that the fish’s collective response interferes with some basic properties of the visual system of birds or even more generally of vertebrates akin to a confusion effect [1,23]. Possible mechanisms include saccadic inhibition [24] triggered by the appearance of waves, birds tracking the wave instead of the fish and/or waves enhancing the contrast with the surrounding water, thereby making fish less conspicuous [25] for 2−3 s after a wave has passed.

Reingold & Stampe [24] showed an inhibition of saccadic eye movements (e.g. a stop of the rapid eye movement between focus phases) in humans after a flash as quickly as 60−70 ms following its onset suggesting a reflex-like oculomotor reaction. Potentially, a wave could have a similar effect on saccades and thus interfere with the ability of a bird to track a fish. Another possibility is that the white appearance of waves leads to contrast reduction which in turn could reduce the ability of a predator to detect a fish at the surface for short time periods of a few seconds after a wave [25]. A third mechanism could be that the bird’s gaze follows the movement of the wave instead of focusing on a given fish and thus occupies the bird’s attention as long as it takes for the next wave to begin. According to a review by Land [26], birds make eye movements, but because they have light heads and highly flexible necks, gaze changes predominantly involve head movements. This has been used in recent studies on visual attention of free-flying homing pigeons [27] and starlings under laboratory conditions [28]. A similar approach applied to predatory birds in our study system should be able to shed more light on where the birds direct their attention during wave movement and thereby help identify the mechanisms that cause long waiting times until the next attack.

Besides the confusion effect, another possibility is that the fish use waves as signals to advertise their detection of predators to avoid attacks. Informing bird predators that they have been discovered could be evolutionarily stable because the birds save the energy to carry out attacks with a low chance of success and fish save the energy to carry out unneeded evasion behaviours [29,30]. Regardless of whether the underlying mechanism is one of confusion or signalling, the question remains why similar numbers of waves at similar frequencies are effective for a heron on a river bank that is just 1 m away and for a kingfisher high up in a tree at a distance of up to 20 m. Clearly, much more information is needed about the sensory perception of predators which are at the receiving end to shed light on this problem.

We like to point out that the current dataset did not reveal a decrease in capture probabilities among the studied bird species as a response towards observed waves, which was previously observed for the kiskadee not included in this study [10]. This discrepancy might be due to the fact that kiskadees are not specialized fish eaters and thus their capture success could be much more prone to the waving of the fish as compared with the four species investigated herein that are all known for their piscivorous diets. Furthermore, the hunting style of kiskadees differs vastly from the four species reported here as kiskadees just enter their beaks into the water not causing multiple waves during their own attacks. Thus, kiskadees may not expect waves after attacks and are thus more affected if (in [10] through experimental manipulation) waves become visible to them.

### 4.2. The fishes’ responses and the role of uncertainty

It is well documented that individual escape responses in prey species can differ markedly with the predator species. For example, vervet monkeys, *Chlorocebus pygerythrus*, flee into the undergrowth if a raptor approaches but climb up into the thin branches of trees if a leopard is coming [31]. In contrast, little information is available on collective prey responses to different threat levels and predators [32,33]. Such information would be needed to potentially shed light on which components of collective antipredator responses are particularly important/effective and therefore intensified at elevated threat levels. Similarly, prey collectives might also show different types of collective behaviours in response to predators with different attack strategies [34].

While we did not find differences in waving behaviour in response to different predator species or their attack styles, we did find that wave number and to a lesser extent, the time between waves increased with threat level from pre- to post-attack and with increasing sizes of the potential predators in the pre-attack context. The enhanced response after an attack could be explained by the fact that a predator attack provides more stimuli than would be available in the pre-attack context where the predator has just arrived on site simply because a visual stimulus is often paired with an acoustic one as the bird’s head hits the water and also causes mechanical waves at the surface. Integrating cues from multiple sensory modalities is a strategy used by many animals for uncertainty reduction, leading to an increase in startle responses in goldfish, *Carassius auratus* [35], for example, and deeper diving responses in sulphur mollies [15]. In our study system, it appears that the fish show a high (or even their maximum) waving response when multiple predator-related cues coincide (as is the case after an attack) regardless of which predator species attacked. It remains an interesting open question whether the short intervals between waves which fish show at these moments represent their physiological limit or whether it is the optimal interval for disrupting the predator’s attack or part of a signal.

Alternatively, one might argue that the potential to evolve different strategies for different predators (with vastly different hunting styles) was too low in our system. After all, birds are their main (and possible only) predators which attack hundreds of times per day, removing large numbers of individuals ([10,14,15], see also capture per bout data in electronic supplementary material, figure S2). Often, many different bird species hunt simultaneously or in short succession [10,14,15], thus making it highly likely for the fish to gather different species-specific cues during the attacks. In addition, members of the poeciliid family are among the model species for fast evolution as they are, for example, known for producing multiple generations per year creating the potential for rapid adaptations [36]. Indeed, poeciliids contain several invasive species and have colonized extreme environments such as caves and sulphur springs because of this adaptive ability [37]. We therefore would argue against a lack of evolvability as an explanation for the similarities in responses towards attacks by different bird predators.

### 4.3. Importance and relevance of our approach

In this study, we investigated the collective responses of one prey species to several different predators, and we tested selected aspects of predator cues (size and colour) in highly replicated experiments to understand whether prey modify their behaviour for different predators or show a largely generic response to all of them. Besides our study that investigated a single study system in the wild, a potential alternative to answering this question (or to our approach) is to take studies from the literature which reported on antipredator behaviours such as selfish herd aggregation [38] or predator inspection [39–43] across different prey and predator species to look for differences and generalities. To the best of our knowledge, such an approach has not been taken so far based on quantifiable collective behaviours in prey and attack and capture rates in predators. And, it would certainly be very interesting to carry out such an analysis. A potential problem of going across different prey species, however, could be that confounding factors are introduced which are hard to control for. For example, the presence or absence of potential refuges in the environment or differences in body morphologies could make such comparisons difficult. These confounding factors could potentially be controlled, however, if a sufficiently large number of such studies is available to estimate their influence systematically.

## Data Availability

All necessary raw data to re-run the analysis have been deposited in the electronic supplementary material, data S2 [44]. In case further information is needed to re-run the analysis, this will be made available by the lead contact upon request.
